# Supplementary figures and images for: Survival trends among people living with human immunodeficiency virus on antiretroviral treatment in two rural districts in Ghana
Source: PLoS One. 2024 Mar 6;19(3):e0290810. doi: 10.1371/journal.pone.0290810 (PMC10917304; doi:10.1371/journal.pone.0290810)

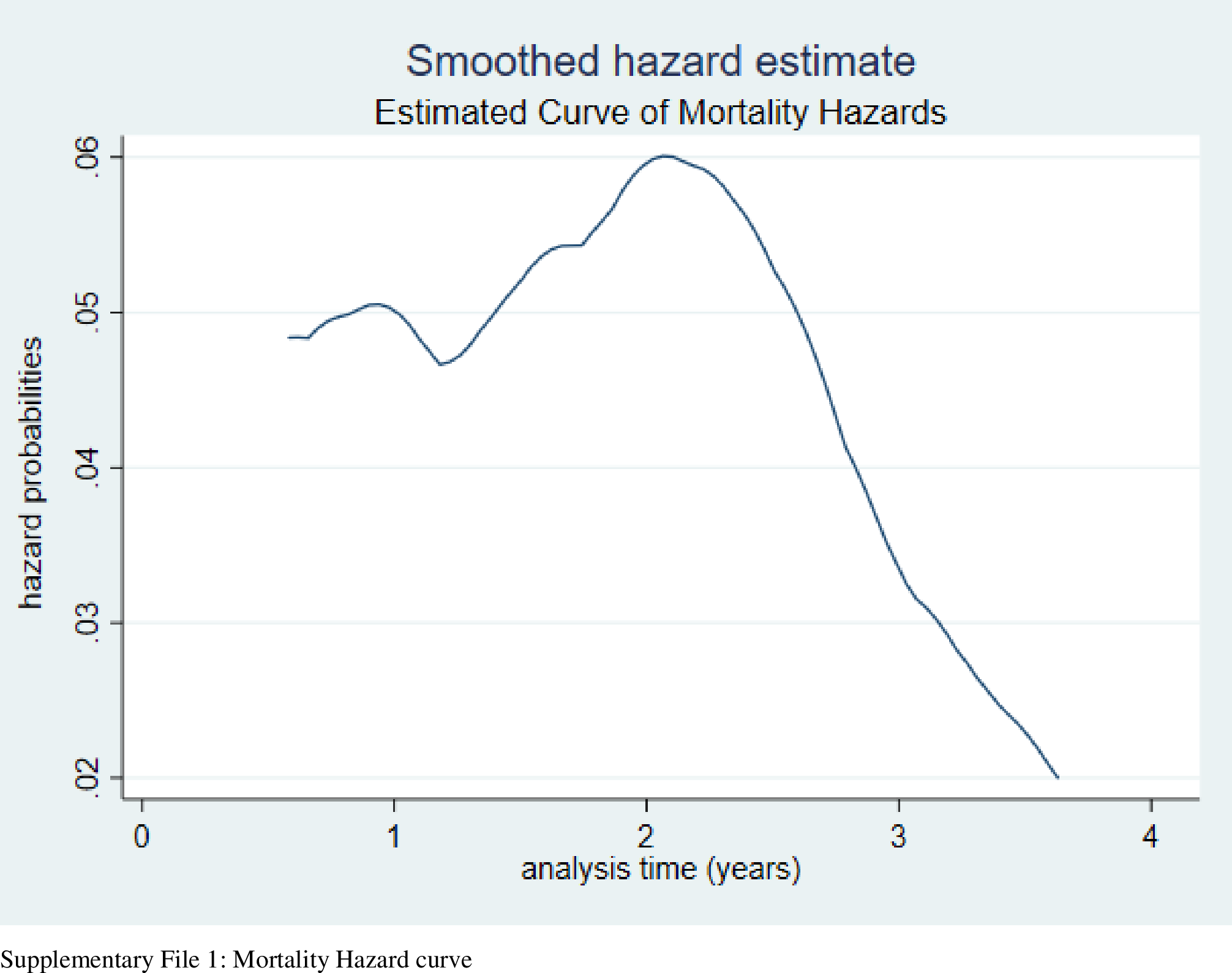

Supplement: S1 File — (TIF) [file pone.0290810.s001.tif]
